# Special Issue on Metabolic Adaptations in Cardiac and Skeletal Muscle during Acute and Chronic Exercise

**DOI:** 10.3390/metabo13091015

**Published:** 2023-09-15

**Authors:** Stephen C. Kolwicz

**Affiliations:** Heart and Muscle Metabolism Laboratory, Department of Health and Exercise Physiology, Ursinus College, Collegeville, PA 19426, USA; skolwicz@ursinus.edu

Research in the field of exercise physiology has evolved dramatically over the last century [1,2]. While early studies primarily focused on physical changes and physiological responses to exercise, the field has emerged to include studies that incorporate advanced technological platforms for genomic, metabolomic, and proteomic analyses [1]. The expansion of the research focus has also provided important knowledge regarding molecular signaling and metabolic pathways. While the benefits of exercise in the treatment of chronic disease are established, the molecular and metabolic adaptations are still not well characterized. This Special Issue of *Metabolites* presents five original research articles from research laboratories in three separate countries, which demonstrate the ability of chronic exercise training to modulate various aspects of metabolism in both humans [3,4] and rodent models [5,6,7]. Moreover, these articles evaluate the potential of exercise training to elicit positive metabolic adaptations in several chronic diseases and conditions.

The study of epigenetic changes in skeletal muscle, particularly via DNA methylation, has been of recent interest in the exercise literature [8]. Garcia and colleagues explored the effect of aerobic exercise training on DNA methylation in human skeletal muscle in sedentary, overweight/obese (i.e., BMI ~ 30.0) subjects [4]. In the study, the 8-week program consisted of moderate (i.e., 60–70% of peak VO_2_) cycle ergometer training for 30–45 min per session. Muscle biopsies of the vastus lateralis were collected before and after training. The exercise training program resulted in significant improvements in aerobic capacity and insulin action, without affecting body composition. Importantly, the authors identified ~100 differentially methylated cytosines (DMCs) in the skeletal muscle in response to exercise training. The findings identified a number of candidate genes that could be important in future research endeavors. 

The hormonal regulation of metabolic processes is critical in the understanding of adaptations to exercise training. Pejon et al. investigated the role of exercise training in mitigating the consequences of aberrant metabolism due to estrogen deficiency [7]. Ovariectomized (OVX) rats completed a swimming training protocol for 30 min/day, five times/week at 80% critical load over a 12-week period. While sedentary OVX rats showed lower expression of CD36 and GLUT4, exercise training in OVX rats resulted in a normalization of both substrate transporters. Overall, this study demonstrated the importance of estrogen in skeletal muscle metabolism as well as the effectiveness of moderate aerobic exercise training to counteract the potential negative metabolic consequences of insufficient estrogen.

In addition to the identification of an appropriate intensity, the frequency (i.e., number of sessions per week) of exercise remains an important consideration. Lavier and associates compared a 7-week, moderately intense, aerobic exercise training program, performed either three times per week or five times per week, on physical performance in an animal model of peripheral artery disease (PAD) [5]. Interestingly, mice with PAD had similar improvements in endurance exercise capacity (i.e., total distance, duration, and speed) on a treadmill test to exhaustion, regardless of whether they exercised three or five times per week. In addition, exercise training in the PAD groups led to the downregulation of genes involved in glucose and fatty acid metabolism without significant effects related to the frequency of training. These results show that three and five times per week of exercise training lead to similar improvements in endurance performance in an animal model of PAD. These findings could be of potential translational value to clinicians working with patients of chronic diseases, particularly PAD. 

While exercise training interventions remain important for patient outcomes, combined interventions including dietary strategies can be critical in the management of chronic diseases, especially obesity and Type II diabetes. The work of Chorell et al. evaluated whether an exercise training program combined with a dietary intervention would improve insulin insensitivity in Type II diabetic patients [3]. Obese, Type II diabetic subjects were placed on a Paleolithic-type diet or a Paleolithic-type diet plus a combined aerobic/resistance training program for 12 weeks. After 12 weeks, both groups had lower body mass index, reduced body fat percentage, and improved peripheral insulin sensitivity. Using metabolomics and lipidomics analyses in blood and skeletal muscle, the authors identified distinct signatures associated with the improvements in insulin sensitivity. The diet-only group demonstrated reductions in the branched-chain amino acid, valine, in both the muscle tissue and the blood. Interestingly, the diet-plus-exercise group had higher diacylglycerol and triacylglycerol in the muscle, consistent with the trained skeletal muscle according to the “athlete’s paradox” [9]. These findings highlight the importance of an in-depth analysis of biochemical pathways to identify possible biomarkers that modulate metabolism in chronic diseases.

O’Neill et al. addressed the value of aerobic exercise training in the treatment of Type I diabetes in mice [6]. Of note, this study performed the investigations in female mice, which are under-utilized in biomedical research. Type I diabetic female mice presented with hyperglycemia, dyslipidemia, and tissue atrophy, similar to findings reported in males. In addition, gene expression analysis suggested that Type I diabetes resulted in reduced ketone body oxidation and elevated fatty acid metabolism. Four weeks of moderate exercise training (60 min per session, 5 times/week) on a motorized treadmill failed to improve the overt phenotype and changes in gene expression. However, the exercise training program did improve insulin sensitivity in Type I diabetic female mice, despite its inability to improve glucose homeostasis.

Collectively, these articles demonstrate the beneficial effects of exercise training in both human and animal models of chronic disease, including obesity, Type I and Type II diabetes, and peripheral artery disease. In these disease models, exercise of a moderate intensity has the potential to induce epigenetic changes, alter metabolic gene expression, improve insulin sensitivity, and increase exercise tolerance. In addition, exercise training may possess the ability to counteract deficiencies in sex hormones. With the use of advanced technologies, including genomic, metabolomic, and proteomic analyses, a deeper mechanistic understanding of the complex metabolic adaptations that result from exercise training will be uncovered. Hopefully, the studies included in this Special Issue will stimulate future work in the field of exercise metabolism.
